# Methodology for the Assessment of the Ecotoxicological Potential of Construction Materials

**DOI:** 10.3390/ma10060649

**Published:** 2017-06-13

**Authors:** Patrícia Rodrigues, José D. Silvestre, Inês Flores-Colen, Cristina A. Viegas, Jorge de Brito, Rawaz Kurad, Martha Demertzi

**Affiliations:** 1 Instituto Superior Técnico, Universidade de Lisboa, Av. Rovisco Pais 1, 1049-001 Lisboa, Portugal; patricia.rodrigues@tecnico.ulisboa.pt; 2 CERIS—Civil Engineering Research and Innovation for Sustainability, Instituto Superior Técnico, Universidade de Lisboa, Av. Rovisco Pais 1, 1049-001 Lisboa, Portugal; ines.flores.colen@tecnico.ulisboa.pt (I.F.-C.); jb@civil.ist.utl.pt (J.d.B.); rawaz.saleem@gmail.com (R.K.); marthademertzi@tecnico.ulisboa.pt (M.D.); 3 Bioengineering Department and iBB—Institute for Bioengineering and Biosciences, Instituto Superior Técnico, Universidade de Lisboa, Av. Rovisco Pais 1, 1049-001 Lisboa, Portugal; cristina.viegas@tecnico.ulisboa.pt

**Keywords:** cement-based, construction materials, ecotoxicology, assessment methodology, environmental risk

## Abstract

Innovation in construction materials (CM) implies changing their composition by incorporating raw materials, usually non-traditional ones, which confer the desired characteristics. However, this practice may have unknown risks. This paper discusses the ecotoxicological potential associated with raw and construction materials, and proposes and applies a methodology for the assessment of their ecotoxicological potential. This methodology is based on existing laws, such as Regulation (European Commission) No. 1907/2006 (REACH—Registration, Evaluation, Authorization and Restriction of Chemicals) and Regulation (European Commission) No. 1272/2008 (CLP—Classification, Labelling and Packaging). Its application and validation showed that raw material without clear evidence of ecotoxicological potential, but with some ability to release chemicals, can lead to the formulation of a CM with a slightly lower hazardousness in terms of chemical characterization despite a slightly higher ecotoxicological potential than the raw materials. The proposed methodology can be a useful tool for the development and manufacturing of products and the design choice of the most appropriate CM, aiming at the reduction of their environmental impact and contributing to construction sustainability.

## 1. Introduction

The construction industry accounts for about 30% of global carbon dioxide emissions, consuming 50% more raw materials than any other economic activity. It is therefore considered an unsustainable sector [1].

Concrete, with a production of about 10 billion tons per year, is the most consumed material on planet Earth [2]. The increase in the production of concrete results in a higher consumption of natural aggregates and cement and, therefore, a higher environmental impact of the construction sector.

Although EUROSTAT [3] data indicate that between 1990 and 2012 the manufacturing and construction industries achieved a 38% reduction in CO_2_ emissions, Portland cement remains the most widely used binder in the construction industry, and is responsible for the most significant part of the concrete environmental impact. According to Huntzinger and Eatmon [4] and JWG N013 Draft TR WI 00350023 [5], cement manufacture accounts for about 5% of the global carbon emissions, being considered the third largest source of emissions in the United States. Their partial replacement by pozzolanic additions such as fly ash (FA), silica fume, or ash from rice husks can, therefore, be a positive contribution towards reducing environmental impacts [6]. The current annual worldwide production of by-products is estimated at about 700 million tons of which 70%, at least, is FA [2]. Thus, to achieve sustainable construction, it is urgent to reduce the production and consumption of cement and natural aggregates.

However, the potential environmental risks associated with changing the conventional composition of construction materials are unknown. There are several materials with some degree of toxicity, not only associated with the environmental impacts from their production, but also with waste produced from this process, which have the potential to be toxic to human health and the environment. It is therefore necessary to evaluate the risks associated with their use [7].

Reducing the environmental impacts of the construction industry is a global concern. Life Cycle Assessment (LCA) allows evaluating the environmental impact of a given product or a service throughout its entire life cycle, from the extraction of raw materials to its rejection and disposal in nature. The environmental impact assessment under LCA can be made using different methods and, consequently, considering different categories of environmental impact. The most recent methods include ecotoxicology in the environmental impact categories on a LCA analysis.

Technical Committee (TC) 350 of the European Committee for Standardization [5] intends, with the design of a standard still in draft (JWG N013 Draft TR WI 00350023—Additional indicators), to clarify the assessment of the environmental impact of construction materials and buildings at the European level, with the introduction of new categories of environmental impact, in particular ecotoxicology, emphasizing the importance of these issues in the products and services of environmental impact assessment. Ecotoxicology is a branch of toxicology that studies the toxic effects caused by natural or artificial substances present in the macroenvironment (air, water, and soil), and in living organisms. Thus, this science may have a strong contribution to the increase of the construction sustainability, since it allows for evaluating the potential environmental risk associated with the materials to be incorporated in construction, even without the need of using LCA. For that purpose, it is necessary to assess the potential ecotoxicity of materials/products by using leaching tests, chemical analyses, and (eco) toxicity tests.

As far as the authors are aware, regarding the assessment of the ecotoxicological potential of materials and raw materials used in the construction sector, regulation is scarce and there is a lack of harmonization among the scientific community. Therefore, the development of risk assessment methodologies for these types of materials is needed. This paper explores the ecotoxicological potential associated with raw materials and construction materials, and proposes and applies an expedient methodology for the assessment of the ecotoxicological potential of raw and construction materials. This methodology allows for assessing the environmental risk arising from the use of new raw and construction materials based on existing laws, such as Regulation (European Commission) No. 1907/2006 (REACH—Registration, Evaluation, Authorization and Restriction of Chemicals) [8] and Regulation (European Commission) No. 1272/2008 (CLP—Classification, Labelling and Packaging) [9].

## 2. Proposed Environmental Risk Assessment Methodology

The methodology for assessing the potential environmental risk of raw and cement-based construction materials for landfilling and ecotoxicological potential proposed in this study was based on the European legislative provisions laid down in the Waste Framework Directive 2008/98/EC [10], the Directive 1999/31/EC on the landfill of waste [11], the Council Decision 2003/33/EC [12] establishing criteria and procedures for the acceptance of waste at landfills, and the REACH and CLP regulations; it is also based on the criteria and evaluation methodology for waste ecotoxicity (CEMWE) proposed by the French Environment and Energy Management Agency [13] and previously adopted by [14] in the chemical and ecotoxicological characterization of ashes.

**Figure 1:**
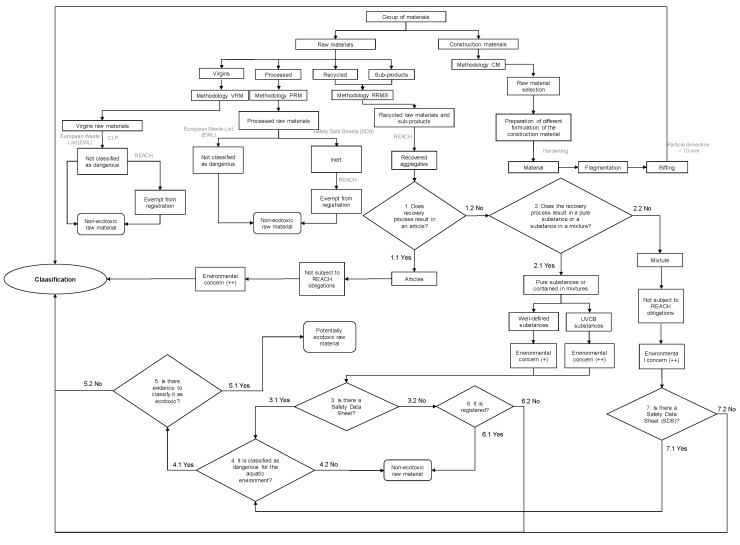
Flowchart for the application of the proposed methodology for different groups of materials.

The methodology for evaluating the ecotoxicological potential (Figure 1) divides the materials in two distinct groups: (a) raw and (b) construction materials. The first one includes materials that are incorporated in cement-based products and is organized into the following subgroups: (i) virgin raw materials; (ii) processed raw materials; (iii) recycled raw materials; and (iv) raw materials resulting from by-products. This grouping is related to the usefulness of the above-mentioned materials in the replacement of Portland cement and natural aggregates (NA), aiming at the production of high performance materials that can contribute to the sustainability of the construction sector. For each group of raw materials, a methodology for evaluating the respective potential of ecotoxicity was defined: methodology VRM (virgin raw materials); methodology PRM (processed raw materials); methodology RRMS (recycled raw materials and sub-products); and methodology CM (construction materials) (Figure 1).

### 2.1. Assessment for Virgin Raw Materials (VRM)

The subgroup of virgin raw materials includes all the materials that have only undergone physical changes (i.e., mechanical processing and eventual sieving), such as NA. Although the reduction of the consumption of natural resources is a key factor in achieving sustainability in the construction sector, this subgroup should not be neglected because of their current use in conventional construction materials. Traditionally, in the manufacturing of cement-based construction materials, NA extracted from quarries of different geological origins are used. Those aggregates are then mechanically processed in order to obtain the desired characteristics.

A proposal to determine the ecotoxicological potential of VRM is made in Figure 1, and is based on the European List of Waste (ELW) [15] in compliance with the Waste Framework Directive [10], and on the legislative provisions of CLP and REACH. This methodology is justified because: waste from the extraction of metallic or non-metallic ores is not identified as hazardous in the ELW; virgin raw materials occur in nature and are not chemically modified, and therefore do not meet the criteria for classification as dangerous substances under CLP, being exempt from compliance with the provisions of registration under REACH. Under these circumstances, it is legitimate to consider that virgin raw materials are not potentially ecotoxic and therefore do not pose a significant risk to the environment.

### 2.2. Assessment of Processed Raw Materials (PRM)

Processed raw materials result from an industrial process that requires a specific quality control, being incorporated in cementitious materials in order to provide them with specific properties. Portland cement and aggregates that are artificially produced by thermal expansion (e.g., expanded clay), and which are produced from natural raw materials, stand out in this group.

The methodology proposed for the determination of the ecotoxicological potential of PRM shown in Figure 1 is similar to the VRM approach although based on different assumptions. Processed raw materials, with the exception of cement, result in inert, lightweight artificial aggregates that do not pose a risk to the environment. This methodology has been developed on the basis of the ELW, REACH provisions, technical datasheets, and safety data sheets (SDS) of artificial aggregates from several companies [16,17].

### 2.3. Assessment of Recycled Raw Materials and Sub-Products (RRMS)

The group of recycled raw materials includes recycled aggregates from the fragmentation, separation, sifting, and eventual washing of Construction and Demolition Waste (CDW), and from other industries such as pre-fabrication. Cement can be partially replaced using by-products with pozzolanic (“pozzolans” without specific identification, FA, silica fume, rice husk ash, metakaolin) or latent hydraulic (blast furnace slag, boiled schist, calcareous fly ash) characteristics.

The methodology defined for classifying the ecotoxicological potential of RRMS was based on the concepts and regulatory provisions set out in REACH and is also presented in Figure 1. For the application of the RRMS methodology, it will be necessary to consider that in the lozenges of the flowchart of Figure 1 only numbered questions of “Yes” or “No” answers were considered (further detailed in Table 1).

The registration obligations in REACH only apply to substances, whether pure, contained in mixtures, or articles. According to this Regulation, RRMS are considered to be aggregates or recovered substances after they cease to be waste under Directive 2008/98/EC. However, for those that have not yet ceased to exist, the obligations arising from REACH do not apply. Therefore, the proposed methodology considers that RRMS used in cement-based materials should be considered as recovered aggregates. In fact, recycled materials and industrial by-products are a major environmental concern given the variability and uncertainty that exist as a result of their unpredictable composition, because they can be UVCB substances (i.e., substances of Unknown or Variable Composition, Complex reaction products or Biological materials) (Figure 1, Table 1) [19,20,21]. The application of the RRMS methodology assumes that, if there is a SDS (Safety Data Sheet) for a particular substance, it is delivered to the downstream user. If there is no SDS, then a document with the necessary information to guarantee the protection of human health and of the environment should be delivered.

### 2.4. Assessment of Construction Materials (CM)

**Figure 2:**
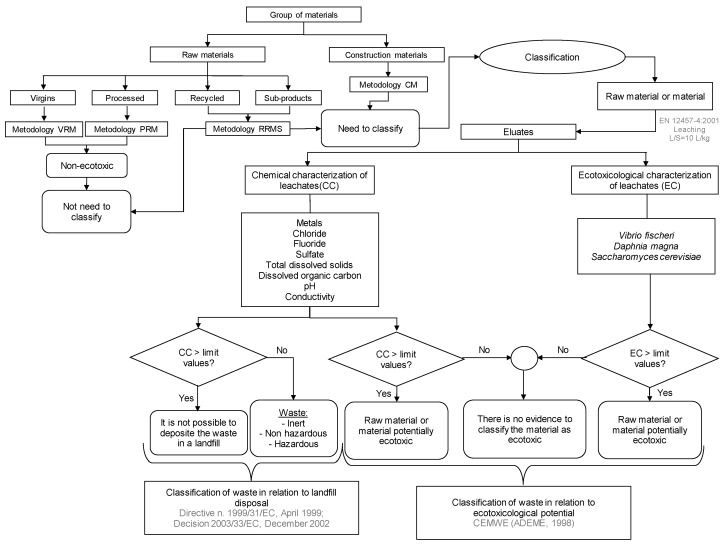
Flowchart representing the methodology for the performance of the chemical and ecotoxicological characterization, and classification of raw and construction materials.

All cement-based materials, such as concrete and mortar, belong to the group of construction materials regardless of the raw materials used. Both the methodology defined in the French proposal CEMWE [13] and the leaching test described in EN 12457/1-4 [22] to comply with Directive 1999/31/EC and Council Decision 2003/33/EC on the landfill of waste are not applicable to monolithic waste but rather to granular solid waste. Despite that, the leaching limit values indicated in the Directive may be applicable to monolithic waste “until specific criteria or criteria are established at the Community level” [11]. Therefore, there is a demand among civil engineers and regulators for methodologies that fit in the above-mentioned documents to evaluate the ecotoxicological potential of construction materials (CM). In this work, a methodology for CM (hereafter named CM methodology) is proposed and represented in Figure 1. After the selection of the raw materials and the fabrication of different formulations of the CMs, it will be necessary to fragment the materials so that they become granular (particles smaller than 10 mm) rather than monolithic [23]. Subsequently, eluates are produced by applying the European Standard leaching test EN 12457/1-4 [22]. The data obtained from both the chemical and the ecotoxicological analysis of the obtained eluates can be compared with those recommended in the regulatory documents mentioned above. Based on these comparisons, it is then possible to classify the CM as defined in Figure 2.

The methodology herein proposed (Figure 1 and Figure 2) assumes that the material is fragmented by grinding or crushing, thus allowing the increment of the contact surface between the material and the solvent used in the leaching procedure. This may lead to an increment in the release of chemical pollutants and, consequently, can result in much higher values of leaching and ecotoxicological potential than under the CM’s normal service conditions. This more conservative approach may represent a worst-case scenario of environmental contamination and/or may correspond to a disposal end-of-life phase of the CM or raw material under study.

### 2.5. Classification Methodology

The classification of materials should be done according to the methodology proposed in Figure 2. The need to classify the materials using this methodology will depend on the pre-existing information about each material under study. When there is not enough data on the raw materials or/and in the case of CM, the procedure for classification begins with the leaching of the raw (or construction) material according to EN 12457-4 [22]. Then, the obtained eluate samples will be subjected to both chemical characterization (CC) and ecotoxicological characterization (EC) (Figure 2).

Regarding the CC, the parameters selected to be determined by chemical analysis (Figure 2) were the ones considered relevant in Directive 1999/31/EC of 26 April 1999, regarding the deposition of waste in a landfill, which coincide at least partly with a number of parameters in the French proposal CEMWE [13]. Thus, the CC will allow the classification of waste in relation to landfilling when the CC values measured in the eluate samples are lower than the limit values set out in the Directive and its associated Council Decision 2003/33/EC. On the other hand, to classify the eluate samples regarding their ecotoxicological potential based on the CC, the obtained chemical values should be compared with the limit values established in the CEMWE French proposal [13] (Figure 2).

Regarding the EC, only short-term ecotoxicity tests were chosen to be performed, because short-term effects represent a more conservative scenario. The choice of the test organisms was made on the basis of the regulatory requirements of the CLP and the REACH and on previous studies reporting the potential ecotoxicity of eluates from bottom/fly ashes [14], of solid waste landfill eluates [24], or of chemical xenobiotics in aqueous solution [25,26]. The results are compared with the limit values set out in the French proposal CEMWE [13] (Figure 2). In this work, three toxicity-tests were selected for the assessment of the ecotoxicity level of the eluate samples obtained from raw materials/CM, as follows: (i) the short-term bacterial bioluminescent test, which uses the marine bacterium *Vibrio fischeri* as a test-organism and can provide a fast evaluation of chemical toxicity. The test measures the light emitted by a standardized suspension of bacterium cells upon 15 or 30 min exposure to the samples in comparison to exposure to a control solution with no toxicant, and a decrease in bioluminescence reflects the magnitude of toxic action. It has wide acceptance among scientists and environment regulators for routine screening of the potential hazard of chemical solutions, sewage effluents, industrial wastewaters, aqueous extracts (e.g., eluates, leachates) of sediment, soil, waste, or ashes, etc. [14,27,28]; (ii) the *Daphnia magna* short-term acute toxicity test, which assesses the inhibition of the mobility of this standard planktonic cladoceran after 24- and/or 48-h exposure to the eluate samples to be tested. This test provides ecotoxicity data relevant for organisms from freshwater aquatic ecosystems, being widely used and recommended at the regulatory level for the testing of industrial or sewage effluents, wastewaters, leachates, and eluates [14,29,30]; and (iii) the 16-h microplate susceptibility test, which measures inhibitory effects of the eluate samples on the growth of the microbial eukaryotic model *Saccharomyces cerevisiae*. This simple, animal-alternative, and relatively inexpensive test system has been proven to enable rapid screening of the potential toxicity of chemicals in aqueous solutions, effluents, and eluates, while being meaningful for experimentally less operative and more costly eukaryotes, like for instance *D. magna* and other aquatic animals [25,26,30].

## 3. Material and Methods

The raw materials chosen for analysis in this study include virgin raw materials (natural, fine, and coarse aggregates), processed (Portland cement), and industrial by-products (FA type F). Regarding the construction materials to be studied, one concrete mix was prepared (B1). The tests carried out within the scope of this study are summarized in Table 2.

### 3.1. Natural Aggregates (NA)

**Figure 3:**
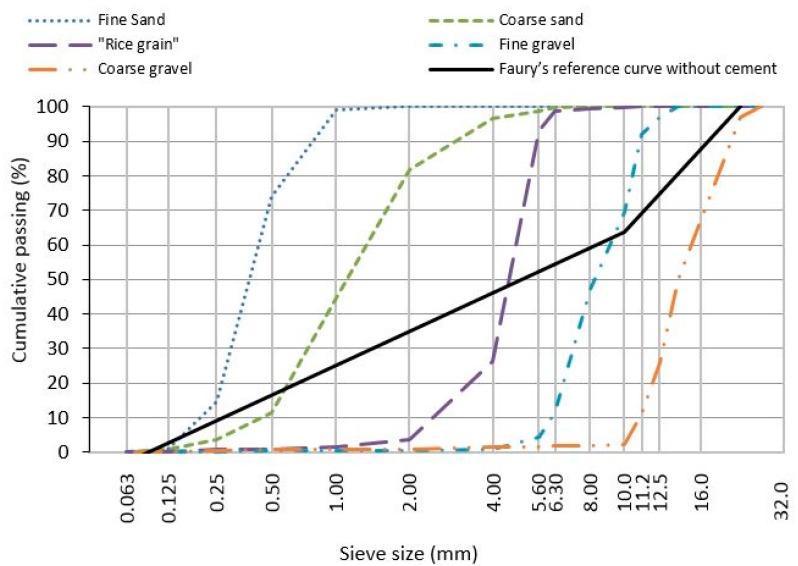
Particles size and distribution of aggregates.

NA are virgin raw materials which were only subjected to physical changes (mechanical processing and possible washing). In this study, two types of fine aggregates and three types of coarse aggregates were used: fine and coarse natural silica river sand and crushed limestone gravels of different sizes (rice grain, fine gravel, and coarse gravel) (Figure 3). The main properties of NA are presented in Table 3. The methodology VRM is used to classify them in terms of their ecotoxicological potential (Figure 1 and Figure 2).

### 3.2. Portland Cement

Portland cement is the most used binder in the construction industry, accounting for the most significant part of the environmental impacts caused by concrete. According to Huntzinger and Eatmon [4], and in accordance with JWG N013 Draft TR WI 00350023 [5], cement manufacturing accounts for about 5% of global carbon emissions and is considered the third largest source of emissions in the United States and is responsible for the poor environmental performance of concrete.

The Portland cement used in this work is CEM I 42.5R (Table 4) and presents a composition with a clinker content of 95% or more.

### 3.3. Fly Ash (FA)

The FA used in this study were produced and supplied by Portuguese companies, have high calcium contents [35], and were classified as type F. Their chemical composition is presented in Table 5. FA are an industrial by-product that results from the burning of pulverized coal in the boilers of thermoelectric power plants.

### 3.4. Cement-Based Materials (Concrete)

In the scope of this study, concrete (B1) with a volume percentage of 100% NA and, by mass, 60% FA and 40% Portland cement was produced. The composition of the concrete per m^3^ was determined according to the method of Faury. The moulds were prepared (washing, without further application of oil), the aggregates were mixed with 2/3 of the total water content for 6 min, then the binder was added with the remaining water, and mixed for 4 more min. After casting, the hardened concrete was cured and covered to prevent water evaporation. The samples were kept in the laboratory for 24 h in order to gain strength to be demoulded. After that, they were placed in a wet chamber until the testing day (28 days) at a constant temperature of 20 ± 2 °C and relative humidity of 100%. The 28-day compressive strength in cubic specimens and the water absorption by immersion and capillarity of this mix are shown in Table 6.

The concrete specimen produced (B1), with the characteristics mentioned and after 28 days of dry chamber curing (relative humidity of 50 ± 5% and 22 ± 2 °C temperature), were fragmented using a jaw crusher in conjunction with a vertical shaft impact crusher, and sieved (mesh 10 mm wide, sieve series 2 (EN 12620 [39]), producing A1 aggregates.

## 4. Results and Discussion (Case Study Application)

This section discusses the methodology used and the results achieved in this study.

### 4.1. Classification of Materials

Natural aggregates (NA) are considered virgin raw materials and, therefore, the application of the proposed methodology (VRM) allows them to be classified as non-ecotoxic raw materials. This result can be supported by the Barbudo et al. [40] study.

According to the REACH regulation, Portland cement is considered a well-defined mixture of several substances ECHA [41], so point 2.2 of the RRMS methodology applies (Figure 1 and Table 1). Therefore, the cement is neither a processed raw material nor a recovered aggregate. With the exception of clinker, which is not subject to registration, the remaining substances are registered. The existence of a SDS for Portland cement [42] indicates that the mixture meets the criteria for the classification of hazardous substances set out in the CLP Regulation. Specifically, the Portland cement’s SDS indicates this material is not considered dangerous to the aquatic environment [42], at least under the light of ecotoxicological data available to date. This result is consistent with the Hillier et al. [23] and Gwenzi and Mupatsi [43] studies.

However, it is noteworthy that concern exists regarding the rise in the water pH that may result from the addition of cement to the water (e.g., washing, percolation), which may become harmful to the aquatic ecosystems under certain circumstances [42,44].

Gwenzi and Mupatsi [43] compared the leaching of heavy metals from coal FA and concrete incorporating coal FA as raw material. They concluded that the incorporation of coal FA at more than 30% of the binder content in concrete should be avoided unless there are studies showing that the environmental risk of such an application is low [44]. To answer this need, the methodology proposed in this study was tested and validated by applying it to a cement-based construction material comprising concrete B1 where FA was incorporated, partially replacing Portland cement as described in the Materials and Methods section.

In the context of REACH, FA are a recovered aggregate considered as a UVCB substance [18]. According to the Product Information Sheet of the FA used in this work [35], it may pose no (or little) concern regarding ecotoxicological effects on the environment [35]. On the other hand, the REACH dossier for “coal ashes (residues)” (REACH Registration number: 01-2119491179-27-0012) establishes predicted-no-effect-concentration values for organisms from freshwater, marine water ecosystems, and soil ecosystems (available online at https://echa.europa.eu/pt/registration-dossier/-/registered-dossier/15573/1). Despite that and because in this work it was intended to test the performance of concrete where Portland cement is partially substituted with FA as binder, it was important to classify this raw material based on the application of the RRMS methodology (Figure 1) to eluate samples prepared as described in Table 2.

The classification of the concrete material B1 regarding its ecotoxicological potential assumed the application of the CM methodology (Figure 1) to the eluate sample obtained from the A1 material (fragmented B1 monolith) as described in Figure 2 and Table 2.

In order to obtain the classification of the FA and A1 material, the CC and EC of the respective eluate samples were carried out; the obtained results of which are presented and discussed below.

#### 4.1.1. Chemical Characterization (CC) of the Eluate Samples of FA and A1

The results of the CC of FA and A1 are summarized in Table 7 and Table 8. The following non-metallic parameters were analysed: pH, Electrical Conductivity; Dissolved Organic Carbon (DOC); Total Dissolved Solids (TDS); Chloride; Fluoride and Sulphate.

Both eluate samples under study were found to be extremely alkaline: FA (pH = 11.8) and A1 (pH = 12.4). The pH value of the FA sample was compared with the results of Moreno et al. [44] and Tsiridis et al. [45]. It is observed that the pH value obtained for the FA sample is closer to the mean value of the results of the work of Tsiridis et al. [45] ($\bar{\mathrm{pH}}$_Tsiridis et al. (2006)_ = 11.5; $\bar{\mathrm{pH}}$_Moreno et al. (2005)_ = 10.6), possibly because the eluates were produced based on the same European standard [22].

The pH value of the A1 eluate sample is common in this type of construction materials; indeed, it is close to the concrete pH value (approximately 12.5) [46]. In both cases, the high pH values are possibly due to the presence of carbonates, oxides, and hydroxides that may be formed during combustion processes [14,43]. Nevertheless, the obtained results suggest that the incorporation of FA in concrete can lead to a slight decrease of the pH of the respective eluate and, thus, to lower possible environmental impacts that may result from the release of leachates/eluates from this type of construction materials into natural waters when, for instance, they are disposed in landfills [43].

The electrical conductivity values varied considerably between the two eluate samples studied, being considerably lower for the FA compared to the A1 eluate (2.63-fold). The conductivity values obtained for the FA were compared with the results of Moreno et al. [44] and Tsiridis et al. [45]. Again, the electrical conductivity value obtained for the FA sample is closer to the average value of the results of the work of Tsiridis et al. [45], which may again be related with the fact that the eluates were produced following the same European standard [22].

The low concentration of dissolved organic carbon (DOC) indicates that both samples are practically free of organic matter. The DOC concentration in the FA eluate sample is lower, since the FA results from a combustion process that causes the reduction of the organic carbon contents that could be present. The concentration of chlorides obtained for the eluate samples are very low when compared to the limit values defined in Directive 1999/31/EC.

The concentration of fluoride analyzed in the samples of FA and A1 does not allow identifying whether its presence is zero or quite reduced, since the limit of detection was not reached. This result makes sense, because these materials do not contain residues with fluoride in their constitution.

Regarding the sulphate concentration, the FA sample shows the highest value of all analyzed samples (4400 mg/kg); in the A1 sample, and the value is close to 30 mg/kg.

The following metallic parameters were analyzed: As, Ba, Cd, Cr, Cu, Hg, Mo, Ni, Pb, Sb, Se, and Zn (Table 8). The elements: Cd, Hg, Ni, and Pb belong to the list of priority substances in the field of water policy (Directive 2008/105/EC); of these, Cd and Hg are considered priority hazardous substances. From this set of metals evaluated in the FA sample, Ba (4.6 mg/kg), Cr (2.5 mg/kg), Mo (10 mg/kg), and Se (4.0 mg/kg) were detected at concentrations above the quantification limits. Comparing the results obtained with the study by Moreno et al. [44], it is observed that the concentration values of the eluated metal parameters of FA are close to the average concentration (determined on the basis of 23 different origin samples of FA). For sample A1 only Ba, Cr, and Mo showed concentrations above the minimum detection limit in some samples. The remaining metal parameters had concentrations of zero or lower than the detection limit. For the A1 sample, the Ba concentration is approximately twice the one recorded for the FA sample. Concentrations of Cr and Mo show decreases of 80% and 94%, respectively, compared to those of the FA sample. It is important to note that the concentration of Se for the samples was zero or below the detection limit. The concentration of this parameter reduced from 95% to 100% when compared to the FA sample.

Based on the CC, the results obtained allow classifying the samples of A1 for landfill as non-hazardous waste and the sample of FA as hazardous. As for the ecotoxicity potential, it was not possible to classify them in terms of chemical characterization, since the limit values defined in the French proposal document CEMWE [13] were not exceeded.

#### 4.1.2. Ecotoxicological Characterization (EC) of the Eluates of FA and A1

**Figure 4:**
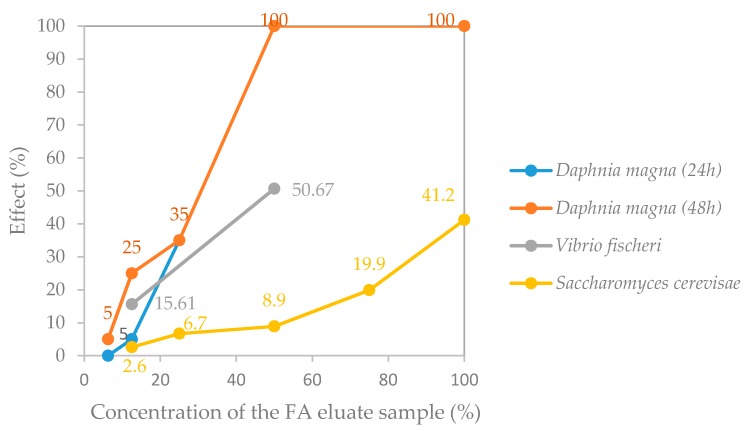
Effect (% of the control) on the test organisms when exposed to different concentrations of the FA eluate sample.

**Figure 5:**
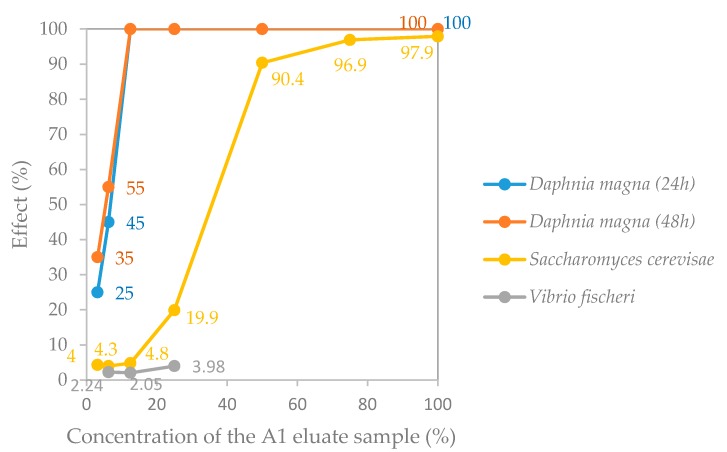
Effect (% of control) on the test organisms when exposed to different concentrations of the A1 eluate sample.

Regarding the EC, dilution series of the eluate samples were subjected to each ecotoxicity test; the obtained concentration-response curves describing the response of each test-organism to the different concentrations of the eluate are presented in Figure 4 and Figure 5. The ecotoxicity indexes estimated from these concentration-response curves for the FA and the A1 eluate samples are presented in Table 9. These data were compared with the limit values referred in the French proposal document CEMWE [13], except for the values recorded for the yeast *Saccharomyces cerevisiae*.

It is worth noting that no corrections were made to the pH of the tested samples since it was intended to simulate environmental exposure conditions close to the ones that may exist in real conditions, and, therefore, such type of intrusive sample manipulation should be omitted [47]. Therefore, the obtained ecotoxicity data (Figure 4 and Figure 5, and Table 9) combine the contributions of possible harmful effects associated not only with chemical components present in the eluate samples but also with the alkaline pH of the eluates (Table 7 and Table 8; discussed in the previous section).

The obtained results show that the bacterium *Vibrio fischeri* and the freshwater crustacean *Daphnia magna* were affected similarly by the FA eluate sample (Figure 4), even though the comparison of the respective toxicity indexes indicates that the *D. magna* ecotoxicity test is slightly more sensitive to the FA eluate sample (48-h mobility EC_50_ = 30.8%) than the *V. fischeri* test (30-min bioluminescence EC_50_ = 49.3%) (Table 9). The yeast was revealed to be less sensitive to the FA eluate sample than the former two ecotoxicity test-organisms (16-h growth EC_50_ > 100%) (Figure 4 and Table 9).

Regarding the A1 eluate sample, Figure 5 shows that both the crustacean and the yeast were affected by this sample, but it did not cause inhibition of the luminescence of the bacterium *Vibrio fischeri* (EC_50_ [30 min] > 100%). Remarkably, the toxicity of the A1 eluate sample for *Daphnia magna* (48-h mobility EC_50_ = 5.5%) was considerably higher than the toxicity measured with the *Saccharomyces cerevisiae* test (16-h growth EC_50_ = 30.2%) (Table 9).

Given these results, according to the ecotoxicity indexes obtained for *V. fischeri* and *D. magna* (Table 9) and their comparison with the ecotoxicity limit values established in the document CEMWE [13], there is no evidence for classifying the FA eluate as potentially ecotoxic. Indeed, the 48-h mobility EC50 for *D. magna* and the 30-min bioluminescence EC50 for *V. fischeri* were higher than the minimum limit value of 10% defined in the CEMWE document [13]. However, given the lower ecotoxicity values obtained for the A1 eluate sample for *D. magna* (48-h mobility EC50 < 10%) (Table 9), this material can be classified as ecotoxic in compliance with the French proposal CEMWE [13].

It should be noted that in other studies reporting ecotoxicity data of leachates/eluates of coal-FA [47] and sludge-FA [14] that were obtained with a leaching standard procedure similar to the one used in this work, the *D. magna* mobility test was also found to be considerably more sensitive than the bacterial bioluminescence test [14,47]. Tsiridis et al. [47] attributed this fact to the low tolerance of the crustacean *D. magna* to the presence of high concentrations of Cr in the samples as well as to a correlation that could exist between the levels of Cu, Ni, and Zn in the samples and their toxicity towards *V. fischeri* bioluminescence [47]. However, in this work, Cr was one of the metals found at higher levels in the FA eluate (2.5 mg∙kg^−1^) but not in the A1 eluate (0.5 mg∙kg^−1^), while the concentrations of Cu, Ni, and Zn were relatively low in both samples (Table 8). Therefore, other factors rather than solely the levels of the referred metals eluated from the FA and the A1 may be influencing the relative biological responses of *D. magna* and *V. fischeri* to the respective eluate samples. Consistently, Lapa et al. [14] suggested that the moderately higher ecotoxicity levels of coal-ash eluate samples towards *D. magna* mobility compared with *V. fischeri* bioluminescence may be related with the *D. magna*’s higher sensitivity to alkaline pH values as high as 11.3 [14]. Consistent with the relative ecotoxicity values obtained in this work, the pH value of the FA eluate (11.8) was close to this value, while the A1 eluate sample was considerably more alkaline (pH 12.4) (Table 9). The high pH values of this type of materials may be due to silicates, alumino-silicates, carbonates, and other oxides produced during the combustion processes [14,43].

Table 10 summarizes the classification obtained with the application of the methodology developed in this study and the parameters considered for this classification. In summary, the eluate of material A1, consisting of 100% NA, 40% Portland cement, and 60% FA (raw materials without evidence of ecotoxicological potential) can be classified as potentially ecotoxic. The potential leaching of metals and other chemicals from both concrete A1 and raw material FA alone were very low for almost all analysed metals, including the ones in the EU list of priority substances in the field of water policy (e.g., Cd, Hg, Pb and Ni) [48]. Nevertheless, regarding landfill deposition and based on the CC alone, the FA eluate was classified as hazardous (Se level) while the eluate of the concrete A1 incorporating FA (at 40%) was considered as non-hazardous (Mo and TDS levels). On the contrary, based on the EC, the former did not show evidence of ecotoxicity while the latter was classified as ecotoxic for *D. magna*. These differences can be due to changes in metal specification in the eluates and/or other parameters not analysed in this work and, importantly, point out the need of complementing the CC with the EC, as suggested before by other authors [14,24]. Based on data presented in this work and from others [14,43], it is thus suggested that the alkaline pH of the FA and A1 granulated concrete may be relevant in this respect and may contribute to possible environmental risks. Such risks can be particularly relevant if eluates or leachates formed from FA or concrete in landfills and/or during building service (e.g., through raining) that may reach freshwater ecosystems leading to water alkalinisation.

## 5. Conclusions

The methodology of environmental risk assessment of construction materials and respective raw materials proposed in this work can have a strong contribution to the construction sector sustainability.

The literature states that cement-based construction materials that raise the most concerns in terms of ecotoxicological risk are those that incorporate recycled aggregates. However, the incorporation of by-products into such materials may also be harmful for human health and the environment. These raw materials, as referred, have a variable chemical composition that is often unknown.

In this context, this paper proposes a methodology for evaluating the ecotoxicological potential of construction materials and their raw materials, considering a conservative scenario, representative of the end of the materials life cycle. This methodology is innovative because it allows for classifying raw materials without resource to CC and EC, i.e., only based on the latest regulations.

The results of CC show that materials formulated with raw materials classified as hazardous (such as FA) may lead to non-hazardous materials (such as A1), based on eluate chemical characterization only. Furthermore, materials with a high ecotoxicological potential, namely A1, which are made of raw materials with no evidence of ecotoxicity (such as NA, Portland cement, and FA) can be formulated from raw materials with evidence of lower ecotoxicity.

The chemical characterization was focused on 19 parameters, 12 of which were metallic, making it possible to classify the materials for the landfill; however, from the 12 metallic parameters analysed in all samples, in the case of the FA sample only 33% were detected at a concentration above the detection limit, and for the A1 sample only 25%. This reduction of the heavy metals leaching between the FA and A1 may be related to the cement's ability to solubilize/stabilize the concentration of heavy metal due to chemical retention processes by incorporation of the elements in the cement matrix, and physical retention by encapsulation.

The ecotoxicological characterization was carried out using acute toxicity tests and allowed the conclusion that, of the three test organisms selected for ecotoxicity tests (*Vibrio fischeri*, *Daphnia magna*, and *Saccharomyces cerevisiae*), the most sensitive in assessing the ecotoxicity of cement-based raw and construction materials is the micro crustacean *Daphnia magna*, which showed the highest levels of sensitivity in all the samples tested. The bioluminescent bacteria shows sensitivity to contact with the FA sample, but it does not show sensitivity to contact with the A1 sample. The yeast *Saccharomyces cerevisiae*, unlike bioluminescent bacteria, shows sensitivity to contact with eluate sample A1, but does not show sensitivity to contact with the FA eluate sample.

In this work, the use of the yeast *S. cerevisiae* as a test organism in the evaluation of the potential ecotoxicity of eluates obtained from materials used in the construction sector is, as far as the authors are aware, innovative. Remarkably, the toxicity data obtained with the yeast-based microplate susceptibility test for the eluates under study were consistent with the ecotoxicity values for the mobility of the standard freshwater model organism *D. magna*, even though the former was moderately less sensitive than the latter. These changes may probably reflect the different complexity of the unicellular *S. cerevisiae* and the crustacean animals as biological systems, with the latter integrating toxicological effects at different levels of biological organization, such as subcellular, cellular, tissue, and organ [25,30]. Results herein obtained thus suggest the yeast may be a relatively good surrogate for preliminary screening and prioritization of the ecotoxicity of this type of materials, before more complex and expensive tests with ecologically more relevant, although experimentally less accessible, animals may be performed [25,30]. The yeast-based test has several advantages as a test system: an alternative to animal experimentation as required by REACH; small scale (<0.1 mL sample required); short exposure time (<24 h); reproducible assessment of many replicates of several samples simultaneously (96-well microplate format); and, easily and inexpensively cultured [25,30].

The application of fly ash in concrete, in partial replacement of Portland cement, leads to low-cost construction materials, contributes to the reduction of the carbon footprint from cement production, and avoids landfilling. It is important to continue carrying out studies on the incorporation of high quantities of fly ash in concrete production, so that this raw material from industrial by-products is used and incorporated in the largest percentage possible.

In the environmental context, the application and validation of the proposed methodology allowed concluding that raw material without clear evidence of ecotoxicological potential but with some ability to release chemicals can lead to the formulation of a CM (e.g., incorporating 40% FA instead of Portland cement) with a slightly lower hazardousness in terms of CC despite a slightly higher ecotoxicological potential than the raw materials. In our view, the latter aspect may be at least partially related with the strongly alkaline pH of the CM eluate, with a pH value slightly higher than the FA eluate, as discussed above.

Thus, it is considered that this methodology can be a useful tool for manufacturers, architects, engineers, and designers, in the development and manufacturing of products. Moreover, it can be useful in the production and design choice of the most appropriate construction materials, aiming at the reduction of the environmental impact and the sustainability of the construction sector.

## Figures and Tables

**Table 1:** Criteria for applying the RRMS methodology.

Methodology RRMS—Application Criteria		
1. “Does the recovery process result in an article?”		
1.1 Yes	A recovery process results in an article when, during production, the shape, surface, or design has been deliberately more important than its chemical composition. Only the substances contained in articles must be registered in REACH. The articles are exempted from registration, so there is a greater environmental concern when this type of raw material is incorporated in construction materials due to the lack of regulation and information that they implicate.	Classify
1.2 No	In the case of a pure substance, a substance contained in a mixture or a mixture.	Proceed to point 2
2. “Does the recovery process result in a pure substance or a substance contained in a mixture?”		
2.1 Yes	The recovery process results in a substance (pure or contained in a mixture) when its chemical composition is more important than the shape, surface, or design. The substances can be well-defined or UVCB (Chemical Substances of Unknown or Variable Composition, Complex Reaction Products, and Biological Materials) and once the former’s chemical composition is defined, it is easy to assess the danger they pose to the environment. For the latter, greater environmental concern is raised, given the variability and uncertainty regarding its chemical composition. This group includes industrial by-products such as slag or fly ash (FA), among others. In fact, the chemical composition of slag and FA is more important for its function than the shape, surface, or design, and they are therefore considered UVCB substances [18].	Proceed to point 3
2.2 No	If the recovery process does not result in an article or a substance, it can result in a mixture. Their use may result in high environmental concern.	Proceed to point 7.
3. “Is there a Safety Data Sheet (SDS)?”		
3.1 Yes	Substances registered under REACH and classified as hazardous under CLP must be accompanied by a Safety Data Sheet (SDS).	Proceed to point 4.
3.2 No	The absence of SDS may mean:	Proceed to point 6.
	(1) A non-hazardous substance and therefore, even if it is registered in REACH, a SDS is not compulsory. This means that it does not meet the criteria for classification of danger for the aquatic environment under the CLP, and that it is a non-ecotoxic raw material; (2) A substance not registered in REACH because it is produced in quantities lower than one tonne per year or that is being used for Research and Development purposes only (exempt from registration). In this case, it is proposed that it is classified (Figure 2).	
4. “Is it classified as dangerous for the aquatic environment?”		
4.1 Yes	The SDS for a specific substance or mixture identifies its classification in relation to the hazards in Section 02. A substance or mixture which is very toxic to aquatic organisms and subject to an acute toxicity test is identified by code H400.	Proceed to point 5.
4.2 No	If SDS clearly indicates that the substance does not cause adverse effects on aquatic organisms, then it may be classified as non-ecotoxic based on the information available to date.	Non-ecotoxic raw material
5. “Is there evidence to classify it as ecotoxic?”		
5.1 Yes	When the information presented in the SDS is clear and it presents data of toxicity indexes (for instance, EC_50_ or LC_50_) for aquatic organisms, this information should be used to classify the ecotoxicological potential of the raw materials to aquatic ecosystems.	Potentially ecotoxic raw material
5.2 No	When the information presented in the SDS does not present the results of EC_50_ or LC_50_.	Classify
6. “Is it registered?”		
6.1 Yes	To identify whether a substance is registered, access the ECHA (European Chemicals Agency) database for substances registered in REACH: echa.europa.eu/information-on-chemicals/registered-substances. If the substance is registered and there is no SDS, it is because the raw material is not considered to be ecotoxic based on the information available so far.	Non-ecotoxic raw material
6.2 No	In the case of an unregistered substance, regardless of the reason, it should be classified (Figure 2).	Classify
7. “Is there a Safety Data Sheet (SDS)?”		
7.1 Yes	As occurs with substances, mixtures must also be accompanied by an SDS when meeting the criteria for classification as a dangerous substance, as defined in the CLP Regulation.	Proceed to point 4.
7.2 No	When there is no SDS for a given mixture, it is necessary to classify it (Figure 2). For dangerous substances and mixtures that are made available or sold to the public, a SDS is not required. However, for this exemption to be valid, the supplier must provide enough data to ensure that the measures are taken to ensure the protection of human health and the environment [18].	Classify

**Table 2:** Test methods used in the present study for the classification of the FA and of the fragmented material A1 (obtained from the concrete material B1).

Test		Location	Methodology
Leaching		Analysis Laboratory of Instituto Superior Técnico (LAIST)	EN 12457-4, using a liquid/solid (L/S) ratio of 10 L/kg [22]
Chemical characterization (CC) of eluates	As, Hg, Sb, Se		Internal method LAIST
	Ba, Cd, Cr, Cu, Mo, Ni, Pb, Zn		ISO 11885:2007
	Chloride, Fluoride, Sulphate		SMEWW 4110 B
	Total Dissolved Solids (TDS)		SMEWW 2540 C
	Dissolved Organic Carbon (DOC)		SMEWW 5310 C
	pH		SMEWW 4500 H + B
	Conductivity		EN 27888 [31]
Ecotoxicological characterization (EC) of eluates	Inhibition of bioluminescence of marine bacterium *Vibrio fischeri*		15 to 30 min exposure in static test [28]
	Inhibition of the mobility of freshwater crustacean *Daphnia magna*		24 and 48 h exposure in static test [29]
	Inhibition of growth of yeast *Saccharomyces cerevisiae* (microplate susceptibility test)	Institute of Bioengineering and Biosciences of Instituto Superior Técnico (IST)	16 h exposure [25,26]

**Table 3:** Properties of NA.

Property	Unit	Standards	Gravel			Sand	
			Coarse	Fine	“Rice Grain”	Coarse	Fine
Loose bulk density	kg/m^3^	EN 1097-6 [32]	1385	1391	1449	1684	1626
Oven dried density	kg/m^3^		2625	2742	2681	2600	2594
Water absorption	%		1.4	1.2	1.0	0.5	0.4
Los Angeles abrasion mass loss	%	EN 1097-2 [33]	28 *			x	x
Shape index	%	EN 933-4 [34]	15.6	18.0	17.1	x	x

* Average value of the coarse and fine gravel.

**Table 4:** Chemical composition and properties of cement.

Chemical Composition	Results (%)	Chemical Composition	Results (%)	Physical Properties	Results
Al_2_O_3_	5.0	MgO	1.3	Flexural strength—28d (MPa)	10.1
CaO	63.5	Na_2_O	0.2	Compressive strength—28d (MPa)	57.7
CaO (L)	1.3	SiO_2_	19.5	Specific gravity (g/cm^3^)	3.1
Cl	0.0	SO_3_	3.3	Residue on the 45 µm sieve (%)	6.2
Fe_2_O_3_	3.3	Loss on ignition	2.4	Final setting (min)	231.7
K_2_O	0.6	Insoluble residue	1.2	Initial setting (min)	161.1

**Table 5:** Physical and chemical properties of the FA provided by the supplier.

Chemical Composition (%)									Physical Properties					
LoI	SiO_2_	Al_2_O_3_	Fe_2_O_3_	CaO	MgO	SO_3_	Na_2_O	K_2_O	Sieve Analysis (Retained %)					Density (kg/m^3^)
									200 µm	90 µm	63 µm	45 µm	32 µm	
3.8	57.8	20.9	7.4	3.6	1.0	0.6	1.0	1.7	0.21	2.92	7.82	14.42	22.48	2300.0

**Table 6:** Characteristics of concrete in the hardened state.

Concrete	Compressive Resistance at 28 Days (MPa) [36]	Water Absorption by Immersion (%) [37]	Water Absorption by Capillarity, 24 h (×10^−3^ g/mm^2^) [38]
B1	23.96	11.0	7.0

**Table 7:** Results of the non-metallic parameters for the FA and A1 eluate samples (abbreviations as in Table 2).

Non-Metallic Parameters							
Materials	pH at 22 °C	Conductivity µS∙cm^−1^	DOC mg∙kg^−1^	TDS mg∙kg^−1^	Chloride mg∙kg^−1^	Fluoride mg∙kg^−1^	Sulphate mg∙kg^−1^
FA	11.8	1672	23.0	6450	<30	<10	4400
A1	12.4	4300	11.0	12,000	19.0	<10.0	<30.0

**Table 8:** Results of the metal parameters obtained for the FA and A1 eluate samples.

Metallic Parameters												
Materials	As	Ba	Cd	Cr	Cu	Hg	Mo	Ni	Pb	Sb	Se	Zn
	mg∙kg^−1^	mg∙kg^−1^	mg∙kg^−1^	mg∙kg^−1^	mg∙kg^−1^	mg∙kg^−1^	mg∙kg^−1^	mg∙kg^−1^	mg∙kg^−1^	mg∙kg^−1^	mg∙kg^−1^	mg∙kg^−1^
FA	<0.4	4.6	<0.1	2.5	<0.5	<0.2	10.0	<0.4	<0.5	<0.4	4.0	<0.5
A1	<0.4	9.0	<0.1	0.5	<0.5	<0.2	0.6	<0.4	<0.5	<0.4	<0.02	<0.5

**Table 9:** Ecotoxicological characterization of the FA and A1 eluate samples.

Material	pH	*Vibrio fischeri* EC_50_ (%) [30 min]	*Daphnia magna*		*Saccharomyces cerevisiae*
			EC_50_ (%) [24 h]	EC_50_ (%) [48 h]	EC_50_ (%) [16 h]
FA	11.8	49.3	30.8	30.8	>100
A1	12.4	>100	6.8	5.5	30.2

**Table 10:** Classification of raw materials and A1 material according to EC Directive 1999/31/EC and Decision 2003/33/EC (procedures and criteria for acceptance of solid waste at landfills) and the French proposal document CEMWE.

Group	Samples	Methodology	EC Decision 2003/33/EC Directive No. 1999/31/CE		CEMWE [13]	
			Classification	Parameter	Classification	End Point
Raw materials	NA	VRM	Inert	-	No evidence of ecotoxicity	n.a.
	Portland cement	RRMS	-	-	No evidence of ecotoxicity	n.a.
	FA	RRMS	Hazardous	Se	No evidence of ecotoxicity	n.a.
Material	A1	CM	Non-hazardous	TDS, Mo	Evidence of ecotoxicity	*D. magna* (48-h mobility)

## Supplementary Files

Supplementary File 1

## Abbreviations

The following abbreviations are used in this manuscript:

CC	Chemical Characterization
CLP	Classification, Labelling and Packaging
CM	Construction Materials
CDW	Construction and Demolition Waste
DOC	Dissolved Organic Carbon
EC	Ecotoxicological Characterization
ELW	European List of Waste
FA	Fly Ash
NA	Natural Aggregates
PRM	Processed Raw Materials
RA	Recycled Aggregates
RRMS	Recycled Raw Materials and Sub-products
SDS	Safety Data Sheet
TDS	Totally Dissolved Solids
UVCB	Chemical Substances of Unknown or Variable Composition
VRM	Virgin Raw Materials
