# Supplementary figures and images for: Methodology for the Assessment of the Ecotoxicological Potential of Construction Materials
Source: Materials (Basel). 2017 Jun 13;10(6):649. doi: 10.3390/ma10060649 (PMC5554030; doi:10.3390/ma10060649)

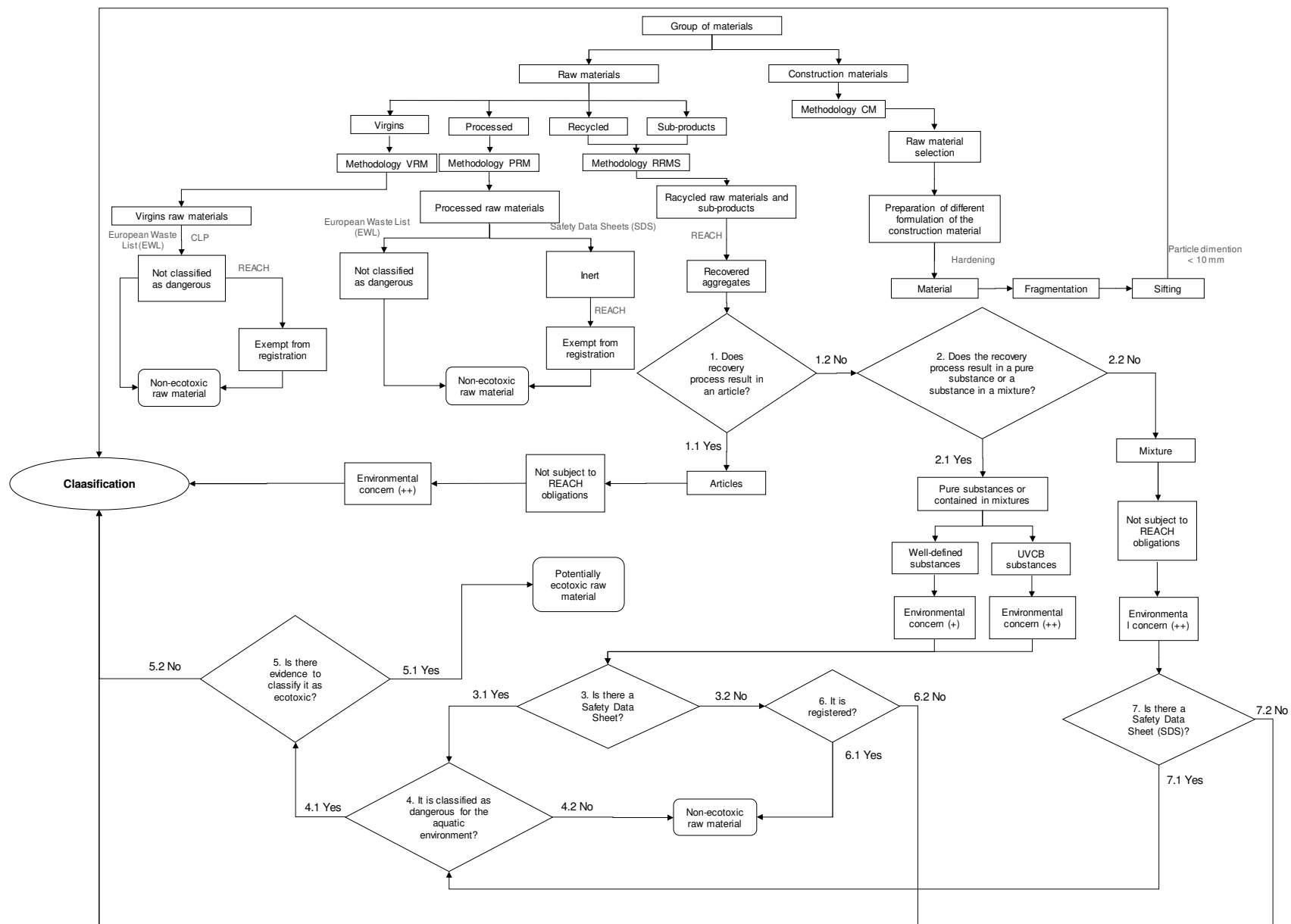

Supplement: Supplementary File 1 [file materials-10-00649-s001.pdf]
